# Low‐Volume Plasma Exchange Combined With a Double Plasma Molecular Adsorption System is Superior to Standard‐Volume Plasma Exchange in improving the MELD Score and Reducing Plasma Consumption in Adult Patients With Acute Liver Failure: A Multicenter Retrospective Cohort Study From Heilongjiang Province

**DOI:** 10.1155/cjgh/6558811

**Published:** 2026-07-13

**Authors:** Yang Liu, Jia-Le Deng, Shu-Xiao Qiu, Di Wu, Hui-Ying Liu, Yu-Jia Tang, Yi-Jin Tang, Zi-Yue Zhang, Qing-Min Meng, Yang Gao, Yan Gao, Kai Kang

**Affiliations:** ^1^ Department of Critical Care Medicine, The Second Affiliated Hospital of Harbin Medical University, Harbin, Heilongjiang, China, hrbmush.edu.cn; ^2^ Department of Critical Care Medicine, The Sixth Affiliated Hospital of Harbin Medical University, Harbin, Heilongjiang, China; ^3^ Department of Emergency, The Fourth Affiliated Hospital of Harbin Medical University, Harbin, Heilongjiang, China, hrbmu.edu.cn; ^4^ Department of Critical Care Medicine, The First Affiliated Hospital of Harbin Medical University, Harbin, Heilongjiang, China, hrbmu.edu.cn; ^5^ Department of Critical Care Medicine, The Fourth Affiliated Hospital of Harbin Medical University, Harbin, Heilongjiang, China, hrbmu.edu.cn

**Keywords:** acute liver failure, artificial liver support systems, standard-volume plasma exchange

## Abstract

**Objective:**

This research aimed to investigate the disparities in improving the model for end‐stage liver disease (MELD) score and plasma volume consumption between standard‐volume plasma exchange (SVPE) and low‐volume plasma exchange (LVPE) combined with the double plasma molecular adsorption system (DPMAS) in adult patients with acute liver failure (ALF).

**Methods:**

This multicenter cohort study retrospectively enrolled adult patients with ALF who were admitted to the designated hospitals and received conventional drug treatment along with one or more sessions of SVPE or LVPE combined with DPMAS during hospitalization. According to the different therapeutic plasma exchange (TPE) methods received during hospitalization, the enrolled patients were subsequently classified into the SVPE group and the LVPE combined with DPMAS group. Thereafter, the aforementioned demographic characteristics, laboratory parameters, their change rates, and plasma consumption were compared between different groups or before and after TPE.

**Results:**

In this multicenter retrospective cohort study, 97 adult patients with ALF were recruited, with a male‐to‐female ratio of 1.06: 1. Subsequently, they were classified into the SVPE group (*n* = 48) and the LVPE combined with DPMAS group (*n* = 49). During hospitalization, they received a total of 97 sessions of SVPE and 91 sessions of LVPE combined with DPMAS. There were significant differences in multiple laboratory parameters before the first SVPE or LVPE combined with DPMAS between different groups, yet the MELD score was not involved. The change rates of multiple laboratory parameters, including the MELD score, and plasma consumption displayed significant differences between the SVPE group and the LVPE combined with DPMAS group.

**Conclusion:**

In terms of improving the MELD score of patients with ALF and reducing the plasma volume, the combined use of LVPE and DPMAS is more effective than SVPE.

## 1. Introduction

Acute liver failure (ALF), also known as fulminant hepatic failure (FHF), typically manifests as a sudden and rapid deterioration of liver function in patients without preexisting liver disease, accompanied by jaundice, coagulation disorders, hepatic encephalopathy (HE), cerebral edema, and even multiple organ dysfunction syndrome (MODS), which is a life‐threatening but potentially reversible emergency [1–3]. Identifying the heterogeneous etiologies of ALF will be conducive to avoiding further deterioration, guiding etiology‐specific treatment, determining potential candidates for liver transplantation (LT), altering disease course, and ultimately affecting clinical prognosis [4, 5]. In recent years, despite there have been tremendous improvement in technology and perioperative management, as the only and most reliable life‐saving procedure available for ALF caused by various etiologies, LT is still seriously hampered by the donor liver scarcity, high expenses, and secondary infection after ALF, especially emergency LT (ELT) [6–8]. Therefore, the use of artificial liver support systems (ALSSs), particularly therapeutic plasma exchange (TPE), to temporarily replace partially or completely lost liver function is instrumental in helping adult patients with ALF survive the most severe clinical stage and in providing sufficient time for spontaneous recovery or serving as a transitional therapy before emergency liver transplantation (ELT), an approach that is also recommended by relevant guidelines [9–11]. In addition, in this clinical situation, its underlying mechanism of action also includes providing metabolic detoxification and synthetic functions, modulating innate and adaptive immunity, improving the liver microenvironment, maintaining liver homeostasis, and stimulating hepatocyte ongoing regeneration [12–14].

The continuous advancements in therapeutic modalities of TPE have expanded the treatment options for adult patients with ALF and acute‐on‐chronic liver failure (ACLF) in daily clinical practice, including standard‐volume plasma exchange (SVPE) and low‐volume plasma exchange (LVPE) combined with double plasma molecular adsorption system (DPMAS), as evaluated in this study [15, 16]. Accumulated evidence has demonstrated that SVPE, LVPE alone, and LVPE + DPMAS are gaining a significant advantage in improving abnormal laboratory parameters, modulating the balance between proinflammatory and anti‐inflammatory cytokines, reducing disease severity, and enhancing survival outcomes in adult or pediatric patients with ALF caused by multiple etiologies [17–20]. The limited availability of plasma, coupled with the high adsorption capacity of DPMAS for toxic metabolites, inflammatory mediators and bilirubin, has driven the transition from SVPE to LVPE + DPMAS, effectively addressing the inherent limitations of standalone TPE and DPMAS [21]. However, to date, no relevant clinical studies have been conducted to investigate the differences in clinical efficacy and plasma consumption between SVPE and LVPE combined with DPMAS (LVPE + DPMAS) in adult patients with ALF.

To address this practical issue, we aimed, for the first time, to investigate the differences in MELD score improvement and plasma volume consumption between SVPE and LVPE + DPMAS in adult patients with ALF in the present study. Our novel findings provide further insight into the distinct advantages and limitations of SVPE and LVPE + DPMAS in the clinical management of adult patients with ALF. These comparisons facilitate the identification of appropriate candidates for each therapeutic modality of TPE and offer a promising strategy to optimize interventions and improve clinical outcomes under the current constraints of an increasingly limited blood supply. This study thus carries significant clinical implications.

## 2. Methods

### 2.1. Study Design

This multicenter cohort study retrospectively enrolled adult patients with ALF who were admitted to the Department of Critical Care Medicine at the First, Second, and Fourth Affiliated Hospitals of Harbin Medical University between January 1, 2020, and June 30, 2025, and who received conventional drug therapy along with one or more sessions of SVPE or LVPE + DPMAS during hospitalization. Demographic characteristics, including gender, age, and weight, HE grade, as well as laboratory parameters—such as white blood cell count (WBC), neutrophil count (NEUT), lymphocyte count (LYMPH), hematocrit (HCT), platelet count (PLT), prothrombin time (PT), prothrombin activity (PTA), international normalized ratio (INR), fibrinogen (FIB), activated partial thromboplastin time (APTT), aspartate aminotransferase (AST), alanine aminotransferase (ALT), gamma‐glutamyl transferase (GGT), albumin (ALB), total bilirubin (TBIL), direct bilirubin (DBIL), indirect bilirubin (IBIL), serum creatinine (SCr), and model for end‐stage liver disease (MELD) score measured within 24 h before and after each session of SVPE or LVPE + DPMAS, and plasma consumption per session—were extracted, compiled, and calculated from the medical records of enrolled patients. Based on these data, the percentage change in each laboratory parameter was calculated after every SVPE or LVPE + DPMAS session. According to the type of TPE received during hospitalization, patients were classified into two groups: the SVPE group and the LVPE + DPMAS group. Subsequently, demographic characteristics, laboratory parameters and their change rates, and plasma consumption were compared between different groups and within groups before and after treatment. This study was conducted in accordance with the principles of the Declaration of Helsinki. The study protocol was reviewed and approved by the Ethics Committees of the First, Second, and Fourth Affiliated Hospitals of Harbin Medical University (IRB number: 2025318, KY2025‐281 and 2025‐ethic review‐241). The medical data required for this study, extracted by the dedicated personnel within our research team from the Medical Record Departments of the First, Second, and Fourth Affiliated Hospitals of Harbin Medical University, had been removed the personal information of the enrolled patients. Therefore, written informed consent from the study participants was waived in compliance with institutional requirements. The study period for all three participating centers was identical, from January 1, 2020, to June 30, 2025.

### 2.2. Study Population

**FIGURE 1:**
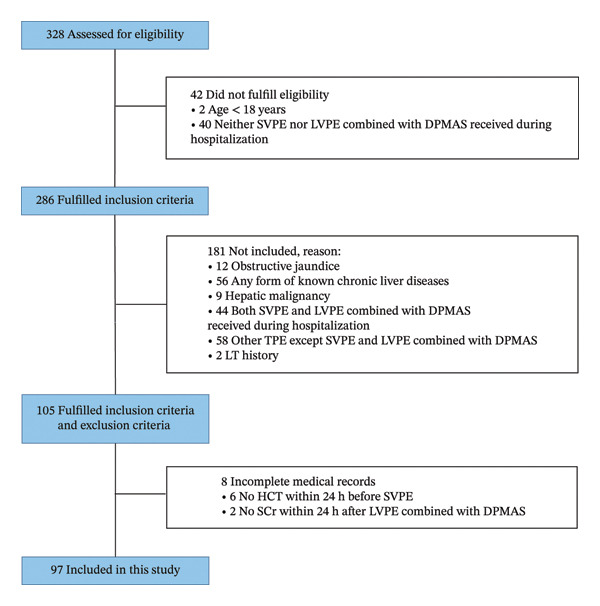
Flowchart of study participants. Abbreviations: SVPE, standard‐volume plasma exchange; LVPE, low‐volume plasma exchange; DPMAS, double plasma molecular adsorption system; TPE, therapeutic plasma exchange; LT, liver transplantation; HCT, hematocrit; SCr, serum creatinine.

The inclusion criteria for this study were as follows: adult patients (aged 18 years or older) with a confirmed diagnosis of ALF who were admitted to the Department of Critical Care Medicine at the First, Second, and Fourth Affiliated Hospitals of Harbin Medical University between January 1, 2020, and June 30, 2025, and who received conventional medical therapy along with one or more sessions of SVPE or LVPE + DPMAS during hospitalization. The exclusion criteria included obstructive jaundice, any known chronic liver diseases, hepatic malignancy, receipt of both SVPE and LVPE + DPMAS during the same hospitalization, use of other TPE modalities beyond SVPE and LVPE + DPMAS, history of LT, pregnancy or breastfeeding, and incomplete medical records (Figure 1).

### 2.3. Diagnosis and Treatment of ALF

In this study, ALF is defined as an acute onset in patients with no previous history of underlying liver disease, accompanied by grade II or above HE (classified by the 4‐grade classification system) within 4 weeks and presenting with the following manifestations: (1) severe gastrointestinal symptoms such as fatigue, anorexia, abdominal distension, nausea, and vomiting; (2) coagulation abnormality, with an INR ≥ 1.5 or PTA ≤ 40%, excluding other causes; (3) progressive elevation of TBIL, in accordance with the Guidelines for the Diagnosis and Treatment of Liver Failure (2024 version) issued by the Infectious Diseases and Hepatology Societies of the Chinese Medical Association [22]. Liver biopsy performed via the transjugular approach remains the gold standard for diagnosing certain patients with challenging clinical presentations [23]. In this study, all enrolled adult patients with ALF received conventional pharmacological treatment and underwent one or more sessions of SVPE or LVPE + DPMAS during hospitalization.

### 2.4. Diagnosis and Grading of HE

In this study, HE is defined as a neuropsychiatric syndrome caused by severe acute or chronic liver dysfunction and/or portosystemic shunting, based on metabolic disturbance and presenting with neuropsychiatric abnormalities of varying severity, in accordance with the Chinese Consensus on the Diagnosis and Treatment of Hepatic Encephalopathy (Chongqing, 2013) issued by the Chinese Society of Gastroenterology and the Chinese Society of Hepatology, Chinese Medical Association. The diagnosis of HE was mainly established based on a clinical background of ALF, cirrhosis and/or extensive portosystemic shunting, the presence of neuropsychiatric abnormalities, auxiliary examinations such as blood ammonia measurement, and the exclusion of other causes of neuropsychiatric abnormalities. Minimal HE was not included in the present study. The severity of HE was classified using the 4‐grade classification system: grade I, mild personality or behavioral changes, impaired attention, sleep disturbance, and possible asterixis; grade II, obvious disorientation, lethargy or apathy, inappropriate behavior, dysarthria and definite asterixis; grade III, marked disorientation, somnolence or stupor with preserved response to stimuli; and grade IV, coma with no response to verbal or external stimuli [24].

### 2.5. Definition of Plasma Consumption in SVPE and LVPE

In this study, plasma consumption in SVPE was defined as the administration of 2–4 L of fresh frozen plasma, whereas in LVPE, it was defined as a volume less than or equal to 0.5 times the patient’s estimated plasma volume [25].

### 2.6. Calculation of Patient’s Plasma Volume

The patient’s plasma volume was calculated based on HCT, gender, and body weight using the following formula: plasma volume = (1 − HCT) × [*b* + (*c* × weight)], where plasma volume is expressed in milliliters (mL) and weight in kilograms (kg). The constants b and c are 1530 and 41 for males, and 864 and 47.2 for females, respectively [26].

### 2.7. Procedure of SVPE and LVPE Combined With DPMAS

A temporary double‐lumen central venous catheter (11, 12, or 14 Fr) was inserted under bedside ultrasound guidance to establish vascular access for SVPE and LVPE + DPMAS. The preflushing solution consisted of 12,500 units of heparin in 3000 mL of normal saline. Given the impaired coagulation function observed in the enrolled adult patients with ALF, no additional heparin was administered during the procedure.

### 2.8. Calculation of MELD Score

The MELD score was calculated using TBIL, INR, and SCr according to the following formula: MELD score = 3.78 × ln (TBIL) + 11.2 × ln(INR) + 9.57 × ln(SCr) + 6.43, where TBIL and SCr are expressed in mg/dL, and ln denotes the natural logarithm.

### 2.9. Data Collection

The medical data required for this study were extracted by designated research team members from the Medical Record Departments of the First, Second, and Fourth Affiliated Hospitals of Harbin Medical University, with all personal identifying information removed. Therefore, written informed consent from the participants was waived in accordance with institutional review board requirements.

### 2.10. Statistical Analyses

In this study, SPSS Version (27.0) was used for statistical analyses. The Shapiro–Wilk test was applied to assess the normality of continuous variables. Non‐normally distributed continuous variables are presented as median (P25, P75), whereas counting variables were expressed as frequency (%). Intergroup comparison of non‐normally distributed continuous variables was performed using the Mann–Whitney *U* test. The Wilcoxon signed‐rank test was used to compare laboratory parameters measured before and after each session of SVPE or LVPE + DPMAS within 24 h. A *p* value < 0.05 was considered statistically significant.

## 3. Results

### 3.1. Intergroup Comparison of Demographic Characteristics and Laboratory Parameters Before the First SVPE or LVPE Combined With DPMAS

A total of 97 adult patients with ALF were enrolled in this multicenter retrospective cohort study, with a male‐to‐female ratio of 1.06: 1, and were subsequently divided into the SVPE group (*n* = 48) and the LVPE + DPMAS group (*n* = 49). These patients underwent a total of 97 SVPE sessions and 91 LVPE + DPMAS sessions during hospitalization. Significant differences were observed between the two groups in several baseline laboratory parameters prior to the first treatment session, including PLT, APTT, AST, ALT, TBIL, DBIL, IBIL, and SCr; however, no significant difference was found in MELD score (Table 1).

**TABLE 1:** Intergroup comparison of demographic characteristics and laboratory parameters before the first SVPE or LVPE combined with DPMAS.

Variable (s)	SVPE group (*n* = 48)	LVPE + DPMAS group (*n* = 49)	*p* value
Gender (female/male)	26/22	21/28	0.362
Age (year)	51.00 (41.50, 58.25)	57.00 (47.00, 63.00)	0.121
Weight (kilogram)	60.00 (55.00, 70.00)	64.00 (57.00, 70.00)	0.403
HE (II/III/IV)	19/24/5	14/27/8	0.446
WBC (× 10^9^/L)	9.56 (6.41, 14.97)	7.89 (6.30, 11.97)	0.318
NEUT (× 10^9^/L)	7.13 (4.33, 11.06)	5.77 (4.22, 9.47)	0.254
LYMPH (× 10^9^/L)	1.25 (0.73, 1.53)	1.10 (0.72, 1.52)	0.951
HCT (%)	29.55 (25.13, 33.73)	33.20 (28.30, 37.50)	0.055
PLT (× 10^9^/L)	84.99 (45.00, 125.50)	120.00 (77.00, 181.00)	0.028
PT (s)	23.10 (18.90, 35.18)	26.60 (20.10, 35.70)	0.468
PTA (%)	31.90 (18.70, 44.63)	30.30 (20.00, 39.00)	0.748
INR	2.09 (1.71, 3.08)	2.20 (1.80, 3.22)	0.525
FIB (g/L)	1.57 (1.22, 2.09)	1.47 (1.02, 1.80)	0.136
APTT (s)	47.15 (34.68, 62.15)	53.40 (44.80, 67.70)	0.032
AST (U/L)	466.10 (165.75, 1607.93)	173.00 (106.00, 373.00)	0.003
ALT (U/L)	530.60 (140.50, 1434.80)	222.00 (88.90, 511.00)	0.025
GGT (U/L)	77.87 (48.00, 145.65)	70.00 (47.00, 113.00)	0.462
ALB (g/L)	31.40 (28.53, 34.20)	30.70 (28.00, 33.10)	0.606
TBIL (μmol/L)	193.65 (96.11, 297.45)	365.70 (297.60, 433.90)	< 0.001
DBIL (μmol/L)	120.90 (45.98, 173.38)	239.00 (175.30, 313.10)	< 0.001
IBIL (μmol/L)	52.00 (24.59, 102.75)	124.70 (86.80, 180.30)	< 0.001
SCr (μmol/L)	116.03 (66.70, 251.38)	71.00 (52.00, 111.00)	0.026
MELD score (point)	27.21 (21.85, 32.03)	25.90 (22.50, 34.06)	0.877

*Note:* WBC, white blood cell count; NEUT, neutrophil count; LYMPH, lymphocyte count; HCT, hematocrit; PLT, platelet count; PTA, prothrombin activity; FIB, fibrinogen, AST, aspartate aminotransferase; ALT, alanine aminotransferase; ALB, albumin; TBIL, total bilirubin; DBIL, direct bilirubin; IBIL, indirect bilirubin; SCr, serum creatinine; MELD, model for end‐stage liver disease.

Abbreviations: APTT, activated partial thromboplastin time; DPMAS, double plasma molecular adsorption system; GGT, gamma‐glutamyl transferase; HE, hepatic encephalopathy; INR, international normalized ratio; LVPE, low‐volume plasma exchange; PT, prothrombin time; SVPE, standard‐volume plasma exchange.

### 3.2. Comparison of Laboratory Parameters Within 24 h Before and After Each Session of SVPE

Significant differences were observed in multiple laboratory parameters when comparing the values 24 h before and after each session of SVPE (Table 2).

**TABLE 2:** Comparison of laboratory parameters within 24 h before and after each session of SVPE.

Variable (s)	Before SVPE (*n* = 97)	After SVPE (*n* = 97)	*p* value
WBC (× 10^9^/L)	9.00 (5.70, 13.59)	9.10 (6.80, 12.44)	0.891
NEUT (× 10^9^/L)	6.78 (4.04, 10.77)	6.96 (4.05, 10.08)	0.689
LYMPH (× 10^9^/L)	1.22 (0.82, 1.55)	1.15 (0.85, 1.62)	0.743
HCT (%)	28.10 (24.50, 32.00)	27.90 (23.00, 32.00)	0.018
PLT (× 10^9^/L)	79.00 (40.00, 108.00)	62.00 (37.00, 100.00)	0.011
PT (s)	22.40 (16.50, 28.10)	19.80 (15.40, 25.10)	< 0.001
PTA (%)	33.20 (24.00, 50.60)	41.70 (28.00, 57.00)	< 0.001
INR	2.05 (1.45, 2.60)	1.75 (1.39, 2.32)	< 0.001
FIB (g/L)	1.50 (1.23, 2.03)	1.53 (1.31, 1.95)	0.301
APTT (s)	43.20 (34.80, 59.00)	40.40 (33.60, 47.57)	0.003
AST (U/L)	257.30 (101.00, 629.10)	134.00 (85.00, 329.00)	< 0.001
ALT (U/L)	206.00 (66.00, 710.00)	126.00 (71.00, 361.70)	< 0.001
GGT (U/L)	60.80 (30.00, 129.10)	46.70 (30.00, 80.85)	< 0.001
ALB (g/L)	32.50 (29.50, 34.90)	32.50 (29.60, 35.10)	0.186
TBIL (μmol/L)	191.20 (100.34, 283.20)	191.40 (109.43, 291.60)	0.151
DBIL (μmol/L)	137.15 (63.20, 179.40)	119.80 (63.20, 177.00)	0.026
IBIL (μmol/L)	68.35 (26.50, 102.70)	68.10 (30.60, 107.90)	0.050
SCr (μmol/L)	110.00 (57.30, 204.00)	131.96 (58.30, 208.00)	0.065
MELD score (point)	24.07 (18.78, 29.88)	22.52 (18.02, 30.57)	0.154

*Note:* WBC, white blood cell count; NEUT, neutrophil count; LYMPH, lymphocyte count; HCT, hematocrit; PLT, platelet count; PTA, prothrombin activity; FIB, fibrinogen; AST, aspartate aminotransferase; ALT, alanine aminotransferase; ALB, albumin; TBIL, total bilirubin; DBIL, direct bilirubin; IBIL, indirect bilirubin; SCr, serum creatinine; MELD, model for end‐stage liver disease.

Abbreviations: APTT, activated partial thromboplastin time; GGT, gamma‐glutamyl transferase; INR, international normalized ratio; PT, prothrombin time; SVPE, standard‐volume plasma exchange.

### 3.3. Comparison of Laboratory Parameters Within 24 h Before and After Each Session of LVPE Combined With DPMAS

Significant differences were observed in multiple laboratory parameters when comparing the values 24 h before and after each treatment session of LVPE + DPMAS (Table 3).

**TABLE 3:** Comparison of laboratory parameters within 24 h before and after each session of LVPE combined with DPMAS.

Variable(s)	Before LVPE + DPMAS (*n* = 91)	After LVPE + DPMAS (*n* = 91)	*p* value
WBC (× 10^9^/L)	7.90 (6.37, 11.31)	8.90 (6.65, 11.94)	0.207
NEUT (× 10^9^/L)	5.79 (4.21, 9.38)	6.73 (4.21, 9.37)	0.054
LYMPH (× 10^9^/L)	1.15 (0.71, 1.63)	1.08 (0.70, 1.56)	0.720
HCT (%)	32.20 (27.00, 38.10)	31.20 (26.30, 35.90)	0.023
PLT (× 10^9^/L)	105.00 (68.50, 179.00)	99.00 (61.50, 166.00)	< 0.001
PT (s)	23.70 (18.40, 34.80)	22.20 (17.20, 27.30)	0.006
PTA (%)	31.60 (20.50, 43.70)	34.80 (26.00, 47.95)	0.002
INR	2.14 (1.67, 3.16)	2.00 (1.53, 2.56)	0.004
FIB (g/L)	1.46 (1.15, 1.81)	1.33 (1.16, 1.72)	0.169
APTT (s)	50.90 (41.15, 62.40)	49.00 (39.70, 60.65)	0.415
AST (U/L)	152.20 (79.50, 297.50)	107.00 (64.00, 197.50)	< 0.001
ALT (U/L)	180.00 (63.50, 362.00)	126.00 (56.00, 258.00)	< 0.001
GGT (U/L)	65.00 (43.50, 99.50)	54.00 (37.00, 82.50)	< 0.001
ALB (g/L)	30.90 (28.95, 34.40)	29.00 (27.00, 32.65)	0.011
TBIL (μmol/L)	347.30 (280.45, 432.95)	302.80 (239.85, 380.40)	< 0.001
DBIL (μmol/L)	221.30 (183.30, 293.10)	182.40 (145.35, 235.35)	< 0.001
IBIL (μmol/L)	121.80 (84.15, 176.80)	113.00 (66.65, 148.22)	< 0.001
SCr (μmol/L)	68.00 (48.00, 110.98)	56.00 (44.00, 106.00)	< 0.001
MELD score (point)	25.39 (20.68, 31.98)	22.26 (18.29, 28.80)	< 0.001

*Note:* WBC, white blood cell count; NEUT, neutrophil count; LYMPH, lymphocyte count; HCT, hematocrit; PLT, platelet count; PTA, prothrombin activity; FIB, fibrinogen; AST, aspartate aminotransferase; ALT, alanine aminotransferase; ALB, albumin; TBIL, total bilirubin; DBIL, direct bilirubin; IBIL, indirect bilirubin; SCr, serum creatinine; MELD, model for end‐stage liver disease.

Abbreviations: APTT, activated partial thromboplastin time; DPMAS, double plasma molecular adsorption system; GGT, gamma‐glutamyl transferase; INR, international normalized ratio; LVPE, low‐volume plasma exchange; PT, prothrombin time.

### 3.4. Intergroup Comparison of the Change Rates of Laboratory Parameters and Plasma Consumption

**FIGURE 2:**
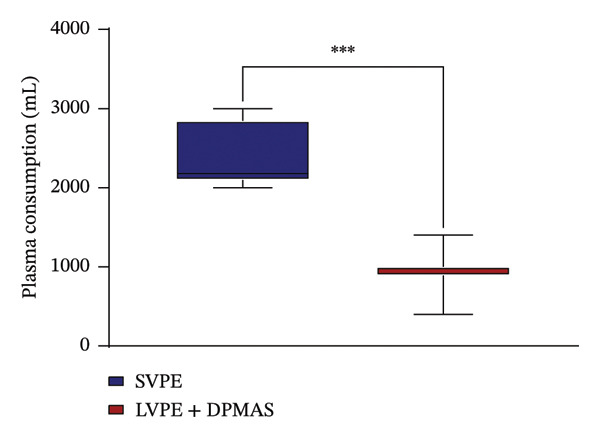
Comparison of plasma consumption between the SVPE group and the LVPE + DPMAS group (^∗∗∗^ *p* < 0.01).

The change rates of multiple laboratory parameters, including MELD score, as well as plasma consumption, showed significant differences between the SVPE group and the LVPE + DPMAS group (Table 4 and Figure 2).

**TABLE 4:** Intergroup comparison of the change rates of laboratory parameters and plasma consumption.

Variable(s)	The SVPE group (*n* = 97)	The LVPE + DPMAS group (*n* = 91)	*p* value
↓ WBC (× 10^9^/L)	2.15 (−26.35, 17.40)	−5.26 (−29.51, 13.14)	0.494
↓ NEUT (× 10^9^/L)	1.54 (−25.08, 21.23)	−9.11 (−35.36, 14.86)	0.229
↑ LYMPH (× 10^9^/L)	−3.47 (−20.55, 30.40)	−1.28 (−22.02, 24.81)	0.649
↑ HCT (%)	−1.99 (−8.05, 3.94)	−2.28 (−7.99, 3.77)	0.873
↑ PLT (× 10^9^/L)	−5.27 (−24.24, 14.18)	−9.92 (−20.07, 6.64)	0.577
↓ PT (s)	6.37 (−2.43, 23.63)	6.62 (−5.93, 19.57)	0.313
↑ PTA (%)	9.21 (−3.14, 44.05)	9.83 (−7.07, 32.04)	0.400
↓ INR	6.55 (−4.61, 23.09)	6.83 (−5.29, 22.79)	0.417
↑ FIB (g/L)	−4.26 (−12.94, 12.11)	−4.44 (−19.77, 12.87)	0.409
↓ APTT (s)	8.86 (−6.02, 23.39)	1.12 (−9.58, 13.72)	0.032
↓ AST (U/L)	33.35 (3.11, 55.11)	23.21 (2.17, 42.68)	0.078
↓ ALT (U/L)	34.65 (11.78, 52.74)	26.95 (12.52, 38.91)	0.059
↓ GGT (U/L)	18.67 (0.00, 36.43)	21.74 (4.48, 36.57)	0.446
↑ ALB (g/L)	0.39 (−5.17, 10.78)	−4.81 (−13.28, 4.80)	0.003
↓ TBIL (μmol/L)	0.48 (−15.66, 16.71)	15.01 (6.14, 27.13)	< 0.001
↓ DBIL (μmol/L)	2.35 (−10.11, 21.19)	20.21 (6.88, 30.79)	< 0.001
↓ IBIL (μmol/L)	−8.16 (−37.95, 11.07)	11.06 (−6.14, 27.69)	< 0.001
↓ SCr (μmol/L)	−4.96 (−24.74, 9.01)	8.20 (−2.23, 22.20)	< 0.001
↓ MELD score (point)	3.39 (−7.38, 11.33)	11.04 (3.83, 18.91)	< 0.001
Plasma consumption	2180.00 (2100.00, 2845.00)	980.00 (890.00, 1000.00)	< 0.001

*Note:* WBC, white blood cell count; NEUT, neutrophil count; LYMPH, lymphocyte count; HCT, hematocrit; PLT, platelet count; PTA, prothrombin activity; FIB, fibrinogen; AST, aspartate aminotransferase; ALT, alanine aminotransferase; ALB, albumin; TBIL, total bilirubin; DBIL, direct bilirubin; IBIL, indirect bilirubin; SCr, serum creatinine; MELD, model for end‐stage liver disease; ↓, Decrease rate; ↑, Increase rate.

Abbreviations: APTT, activated partial thromboplastin time; DPMAS, double plasma molecular adsorption system; GGT, gamma‐glutamyl transferase; INR, international normalized ratio; LVPE, low‐volume plasma exchange; PT, prothrombin time; SVPE, standard‐volume plasma exchange.

## 4. Discussion

To the best of our knowledge, this study represents the first preliminary effort to investigate the differences in clinical efficacy and plasma consumption between SVPE and LVPE + DPMAS in adult patients with ALF. The MELD score and its derived indices, which are well‐established predictive and prognostic tools for patients with ALF, have been widely applied to predict the occurrence of ALF, stratify disease severity, estimate the short‐term mortality—with or without TPE—and assess outcomes following living donor LT, thereby identifying potential candidates for LT [27–31]. From the results of the intergroup comparison of demographic characteristics and laboratory parameters before the first session of SVPE or LVPE + DPMAS, significant differences were observed in multiple laboratory parameters related to coagulation, liver, and renal function between different groups; however, the MELD score was comparable (*p* = 0.877). Each session of SVPE was associated with significant improvements in PT, PTA, INR, APTT, AST, ALT, GGT, and DBIL, as well as a notable decline in HCT and PLT (all *p* < 0.05), indicating that SVPE can not only ameliorate coagulation and hepatic function in adult patients with ALF but may also induce certain adverse effects. Nevertheless, the nonsignificant change in MELD score (*p* = 0.154) within 24 h before and after each SVPE session suggests that SVPE alone may not significantly alter clinical prognosis in these patients.

In contrast, each session of LVPE + DPMAS exerted a positive effect on PT, PTA, INR, AST, ALT, GGT, TBIL, DBIL, IBIL, Scr, and MELD score and played a negative role in HCT, PLT, and ALB (all *p* < 0.05), indicating that LVPE + DPMAS alone may improve clinical prognosis in adult patients with ALF by restoring coagulation, hepatic, and renal function. Based on the intergroup comparison of the change rates of laboratory parameters and plasma consumption, LVPE + DPMAS demonstrated significant advantages in the reduction rates of TBIL, DBIL, IBIL, Scr, and MELD score, as well as lower plasma consumption, whereas SVPE showed superior performance in improving APTT reduction rate and ALB increase rate (all *p* < 0.05). These findings suggest that SVPE and LVPE + DPMAS differ in their therapeutic emphasis regarding the alleviation of abnormal laboratory parameters and plasma consumption, providing guidance for the individualized selection of appropriate TPE modalities tailored to the specific clinical profile of adult patients with ALF in routine clinical practice. Specifically, LVPE + DPMAS is the preferred option for adult ALF patients whose condition is predominantly characterized by decompensated liver and kidney function, whereas SVPE is prioritized for those with severe coagulopathy and hypoproteinemia. In adult patients with ALF who develop MODS, the combined application of multiple therapeutic modalities of ALSSs may provide clinical benefits [32].

Although the optimal therapeutic modality of ALSSs for adult patients with ALF is still controversial, there is increasing recognition that there may be more than one correct approach, which is exemplifies of “one size does not fit all” in the clinical management of these patients. Adopting appropriate therapeutic modalities or combinations of ALSSs tailored to the individual clinical profile of each adult patient with ALF across diverse clinical settings is a key direction in future development. Our study has conducted a preliminary investigation and established a foundational theoretical framework and practical experience in this domain, with particular emphasis on the comparative effects of SVPE versus LVPE + DPMAS on MELD score improvement and plasma volume consumption in adult patients with ALF.

Despite the positive findings, several limitations remain in the present study. First, although adult patients with ALF were enrolled from the three largest tertiary Class‐A comprehensive hospitals in Heilongjiang Province, the most northeastern province of China, over the past five and a half years, the overall sample size is relatively small. Consequently, the nature of a retrospective study with a limited sample size undermines the reliability and generalizability of the conclusions. Second, although the MELD score was comparable between groups in the baseline comparison of demographic characteristics and laboratory parameters before the first session of SVPE or LVPE + DPMAS, significant differences in multiple laboratory parameters related to coagulation, liver function, and renal function between different groups may have influenced the study outcomes. Third, our novel findings are applicable exclusively to adult patients with ALF who underwent conventional pharmacological therapy and one or more sessions of SVPE or LVPE + DPMAS during hospitalization and cannot be generalized to other patient populations with similar clinical conditions. Finally, these conclusions require further validation and refinement through more high‐quality and large‐sample randomized controlled trials (RCTs) conducted in real‐world clinical settings.

## 5. Conclusion

In conclusion, this study provides the first evidence that LVPE + DPMAS is superior to SVPE in improving MELD score and reducing plasma volume consumption among adult patients with ALF. These findings support the individualized selection of TPE modalities tailored to each patient’s unique clinical profile, thereby enhancing clinical outcomes in the context of an increasingly constrained blood supply. As a preliminary investigation, our results highlight the need for further validation through well‐designed, large‐sample, high‐quality RCTs in real‐world clinical settings.

**Nomenclature**
SVPE: Standard‐volume plasma exchange
LVPE: Low‐volume plasma exchange
DPMAS: Double plasma molecular adsorption system
TPE: Therapeutic plasma exchange
LT: Liver transplantation
WBC: White blood cell count
NEUT: Neutrophil count
LYMPH: Lymphocyte count
HCT: Hematocrit
PLT: Platelet count
PT: Prothrombin time
PTA: Prothrombin activity
INR: International normalized ratio
FIB: Fibrinogen
APTT: Activated partial thromboplastin time
AST: Aspartate aminotransferase
ALT: Alanine aminotransferase
GGT: Gamma‐glutamyl transferase
ALB: Albumin
TBIL: Total bilirubin
DBIL: Direct bilirubin
IBIL: Indirect bilirubin
SCr: Serum creatinine
MELD: Model for end‐stage liver disease

## Author Contributions

Yang Liu, Jia‐Le Deng, Shu‐Xiao Qiu, Di Wu, Yang Gao, Yan Gao, and Kai Kang conducted the literature search, conceived and designed the study, performed statistical analysis, interpreted and discussed the results, and were responsible for manuscript preparation, revision, and final approval. Hui‐Ying Liu, Yu‐Jia Tang, Yi‐Jin Tang, Zi‐Yue Zhang, and Qing‐Min Meng contributed to the literature search, data acquisition and compilation, statistical analysis, analysis and discussion of results, and manuscript drafting. Yang Liu, Jia‐Le Deng, Shu‐Xiao Qiu, and Di Wu contributed equally to this work.

## Funding

This study received financial support from the National Natural Science Foundation of China (No. 82372172), the Key Research and Development Plan Project of Heilongjiang Province (No. GA23C007), the Cultivation Project of Heilongjiang Provincial Natural Science Foundation (No. JJ2024LH2132), and the Yijiayi Medical Science Research Fund (No. 2024‐KY003).

## Disclosure

All authors reviewed and approved the final version of the manuscript.

## Ethics Statement

The study protocol was reviewed and approved by the Ethics Committees of the First, Second and Fourth Affiliated Hospitals of Harbin Medical University (IRB number: 2025318, KY2025‐281 and 2025‐ethic review‐241). The medical data required for this study, extracted by the dedicated personnel within our research team from the Medical Record Departments of the First, Second, and Fourth Affiliated Hospitals of Harbin Medical University, had been removed the personal information of the enrolled patients. Therefore, written informed consent from the study participants was waived in compliance with institutional requirements.

## Conflicts of Interest

The authors declare no conflicts of interest.

## Data Availability

The authors are fully committed to provide the raw data underlying the conclusions of this article without reservation.
